# Editorial: Mathematics for Healthcare as Part of Computational Medicine

**DOI:** 10.3389/fphys.2018.00985

**Published:** 2018-07-24

**Authors:** Krasimira Tsaneva-Atanasova, Vanessa Diaz-Zuccarini

**Affiliations:** ^1^Department of Mathematics and Living Systems Institute, University of Exeter, Exeter, United Kingdom; ^2^EPSRC Centre for Predictive Modelling in Healthcare, University of Exeter, Exeter, United Kingdom; ^3^Multiscale Cardiovascular Engineering Group, Department of Mechanical Engineering, University College London, London, United Kingdom

**Keywords:** mathematical modeling, computer simulation, precision medicine, patient specific modeling, digital health

Appropriate mathematical tools and methodologies are critical for ensuring robust and reliable computational model predictions based on medical and healthcare data in the era of the digital health revolution (Duggal et al., 2018). Patient-specific approaches are being increasingly pursued, with simulations benchmarked by clinical data (e.g., brain activity recordings; Breakspear, 2017) obtained in non-invasive manner on individual level (e.g., resting state; Spetsieris et al., 2015). Precision Medicine, although not a new concept, is gaining momentum (Hodson, 2016) powered by the ever increasing volume of patients data (Colijn et al.). Quantifying patient similarity is an important challenge that is critical in predicting patients' disease trajectories (Sharafoddini et al., 2017). In an opinion article (Brown) patient similarity concept has been introduced as a paradigm shift in optimizing personalisation of patient care.

Applications of mathematics in healthcare are achieving unprecedented growth at vertiginous speed in a vast number of areas. Mental health presents a formidable challenge in our modern society and computational psychiatry has recently emerged (Huys et al., 2016) as a field combing computational models and patients' data in an attempt to enhance the prognosis, diagnosis and treatment of mental health conditions. This special issue offers an example (Wong et al.) of statistical learning in the model space for Attention-Deficit Hyperactivity Disorder medication response prediction at individual patient level. Mental health is intimately related to neurological diseases and brain modeling for neurological disease treatments (Rubin, 2017) has found applications to setting deep-brain stimulation parameters in Parkinson's disease treatment (Mandali et al.).

Non-negligible effort is currently being devoted to capturing disease progression, a real challenge in this field. Disease progression modeling involves simulations of disease evolution based on available biomarkers or other time-dependent measures of disease status. This is particularly important in the case of chronic (non-communicable) diseases as demonstrated in this special issue in the case of hypertension (Wang et al.) as well as type 1 diabetes (Wedgwood et al.). Chronic diseases are often extremely complex leading to computational models formulated in high-dimensional space, which poses a challenge for characterizing the pathways of disease progression or patient-specific disease progression trajectories as discussed in Colijn et al. Computational cardiology encompasses mathematical modeling and computer simulation of dynamical processes in the heart and the cardiovascular system in health and disease (Trayanova et al., 2012). An example of an application of computational cardiology is the use of subject-specific computer models to predict neointimal hyperplasia in vein grafts (Donadoni et al.). Computational cardiology applications could help in improving clinical decision support systems in cardiac ablation therapy for example (Green et al.). Moreover, the use of collections of patient-specific models could provide a tool for pre-clinical and clinical assessment of disease pathology such as atherosclerosis and associated calcification (Alimohammadi et al.).

Computational oncology broadly refers to computational modeling and simulations of biological process underlying tumors' development and progression as well as cancer therapy including cancer biomarkers and drug effects (Barbolosi et al., 2016). An example of computational modeling of signaling pathways involved in multiple myeloma is presented in Kendrick et al. whereas (Iuliano et al.) presents a network-based statistical methodology for cancer biomarker selection. Computational and mathematical pharmacology is becoming increasingly relevant for drug development. Mechanistic models has become more and more widely used and our understanding of the models' qualitative and quantitative behavior has improved (Krzyzanski and van Hasselt, 2018). There are outstanding challenges, however, associated with parameter identifiability of pharmacodynamics models (Janzén et al.) as well as the estimation of drug absorption profiles *in-vivo* (Trägårdh et al.).

Robust quantitative methods for identifying biologically/physiologically relevant computational model parameters from experimental data are critical for the successful applications of computational medicine in precision healthcare (Colijn et al.). This special issue presents several examples of such methodological developments in the case of quantifying: the biomechanical properties of human gallbladder (Li et al.); the forces involved in abrasion damage to skin (Jayawardana et al.); and intracellular calcium signals (Mackay et al.).

Infectious disease modeling including the underlying mechanisms is becoming increasingly important in the face of the anti-microbial resistance and its associated clinical and public health burden (de Kraker et al., 2016). Grasping the complexity of host-pathogen interactions remains a challenge and mathematical modeling and analysis could help designing appropriate disease management strategies at patient-specific level (Domínguez-Hüttinger et al.) that are necessary for implementation of precision healthcare (Colijn et al.) as well as to inform public policies related to vaccination, for example see (Hamami et al.).

By no means the topics included in this special issue are exhaustive. They are rather indicative of a wider range of problems specific to computational medicine that not only can be tackled by available mathematical approaches but also inspire the development of novel tools and techniques. Examples of methods that have not been included are machine learning and artificial intelligence for electronic health records analysis and usage (Callahan and Shah, 2018). We hope that future Frontiers Research Topics will contain an increasing number of contributions within the scope of mathematics for healthcare as part of computational medicine.

## Author contributions

KT-A and VD-Z conceived and wrote the editorial.

### Conflict of interest statement

The authors declare that the research was conducted in the absence of any commercial or financial relationships that could be construed as a potential conflict of interest.
